# Magnetic seizure therapy reduces suicidal ideation and produces neuroplasticity in treatment-resistant depression

**DOI:** 10.1038/s41398-018-0302-8

**Published:** 2018-11-23

**Authors:** Yinming Sun, Daniel M. Blumberger, Benoit H. Mulsant, Tarek K. Rajji, Paul B. Fitzgerald, Mera S. Barr, Jonathan Downar, Willy Wong, Faranak Farzan, Zafiris J. Daskalakis

**Affiliations:** 1 Temerty Centre for Therapeutic Brain Intervention, Centre for Addiction and Mental Health, University of Toronto, Toronto, ON Canada; 2 Campbell Family Mental Health Research Institute, Centre for Addiction and Mental Health, Department of Psychiatry, University of Toronto, Toronto, ON Canada; 3 Epworth Healthcare and Monash Alfred Psychiatry Research Centre, Alfred and Monash University Central Clinical School, Melbourne, VIC Australia; 4 Department of Psychiatry, University Health Network, University of Toronto, Toronto, ON Canada; 5 Department of Electrical and Computer Engineering, University of Toronto, Toronto, ON Canada

## Abstract

Therapeutic seizures may work for treatment-resistant depression (TRD) by producing neuroplasticity. We evaluated whether magnetic seizure therapy (MST) produces changes in suicidal ideation and neuroplasticity as indexed through transcranial magnetic stimulation and electroencephalography (TMS-EEG) of the dorsolateral prefrontal cortex (DLPFC). Twenty-three patients with TRD were treated with MST. Changes in suicidal ideation was assessed through the Scale for Suicidal Ideation (SSI). Before and after the treatment course, neuroplasticity in excitatory and inhibitory circuits was assessed with TMS-EEG measures of cortical-evoked activity (CEA) and long-interval cortical inhibition (LICI) from the left DLPFC, and the left motor cortex as a control condition. As in our previous report, the relationship between TMS-EEG measures and suicidal ideation was examined with the SSI. Results show that 44.4% of patients experienced resolution of suicidal ideation. Based on DLPFC assessment, MST produced significant CEA increase over the frontal central electrodes (cluster *p* < 0.05), but did not change LICI on a group level. MST also reduced the SSI scores (*p* < 0.005) and the amount of reduction correlated with the decrease in LICI over the right frontal central electrodes (cluster *p* < 0.05; rho = 0.73 for Cz). LICI change identified patients who were resolved of suicidal ideation with 90% sensitivity and 88% specificity (AUC = 0.9, *p* = 0.004). There was no significant finding with motor cortex assessment. Overall, MST produced significant rates of resolution of suicidal ideation. MST also produced neuroplasticity in the frontal cortex, likely through long-term potentiation (LTP)-like mechanisms. The largest reduction in suicidal ideation was demonstrated in patients showing concomitant decreases in cortical inhibition—a mechanism linked to enhanced LTP-like plasticity. These findings provide insights into the mechanisms through which patients experience resolution of suicidal ideation following seizure treatments in depression.

## Introduction

Major depressive disorder (MDD) is a debilitating mental illness that is associated with a 2.3 times increase in suicidal ideation relative to the general population^1^. More than a third of patients with MDD do not respond to two or more separate trials of antidepressants^2^ and this condition is referred to as treatment-resistant depression (TRD). Electroconvulsive therapy (ECT) is one of the most effective treatments for patients with TRD and it can rapidly reduce suicidal ideation^3^. However, the use of ECT is limited by the cognitive side effects associated with its use.

Magnetic seizure therapy (MST) is a new and promising intervention for patients with TRD^4^. With MST, a therapeutic seizure is triggered by induced currents from time varying magnetic fields. Compared to ECT, MST has more benign cognitive side effects^5^ due to differences in the intensity and spread of the stimulating electrical current^6^. ECT delivers electrical current directly to the scalp, but most of the current is shunted through the scalp due to the skull acting as an insulator that prevents current from passing to the brain. In contrast, the magnetic field from MST is not affected by the skull and induces electrical current inside the targeted brain region, allowing for more focused stimulation^6^.

While MST has been shown to be an effective treatment for TRD^7^, its mechanism of action has yet to be fully determined. Insights into the mechanism can be obtained from previous studies on ECT, which suggest that neuroplasticity may be central to the therapeutic benefit of seizure therapies. According to this neurotrophic theory, activation of large brain networks that occurs during a seizure produces neuroplasticity that can reverse deficits found in MDD, including decreased hippocampal volume^8^, reduced prefrontal gray matter thickness^9^, and compromised white matter integrity^10^. Indeed, neuroimaging studies have shown that treatment with ECT is associated with increased hippocampal and amygdala volume^11^, increased prefrontal and cingulate cortical thickness^12^, and increased fractional anisotropy of anterior cingulum white matter tracts^13^.

The use of transcranial magnetic stimulation combined with electroencephalography (TMS-EEG) is a means to noninvasively assess neuroplasticity in humans. TMS-EEG assessments are done through stimulation of a targeted brain region and measuring the associated brain response. For patients with TRD, a key target region is the dorsolateral prefrontal cortex (DLPFC), which is essential for executive functions such as attention, cognition, and working memory^14^ and has been implicated with the pathophysiology and treatment of MDD^15^. For quantifying neuroplasticity, a candidate measure is cortical-evoked activity (CEA), which is defined as the area under the curve of the rectified single pulse TMS-evoked potential (TEP)^16^. Since CEA accounts for both the peaks and troughs of the TEP waveform, it captures the brain’s ability to respond to a stimulus, and hence its capacity for neuroplasticity. Increased CEA in the prefrontal cortex has been previously observed using a TMS paradigm known as paired associative stimulation (PAS)^16^, which demonstrated long-term potentiation-like (LTP-like) plasticity. Likewise, ECT can also produce LTP-like plasticity in the brain that is reflected as an increase in TEP fluctuations^17^. Given that LTP has been implicated in MDD pathophysiology and that antidepressant treatment enhances LTP^18^, it is possible that MST exerts its therapeutic effects by producing LTP-like plasticity in the cortex, which would be reflected by an increase in CEA near the site of stimulation. Moreover, since previous findings have shown that suppression of GABAergic inhibition leads to improved LTP^19^, we also evaluated if measures of GABAergic inhibition decreases as a result of a treatment course of MST. The selected TMS-EEG measure was long-interval cortical inhibition (LICI), which has shown reliability for characterizing GABAergic neurotransmission^20^.

To determine whether neurophysiological change is associated with clinical improvement, we focused on suicidal ideation as the clinical outcome. Suicidal ideation is prevalent amongst patients with severe MDD and can have morbid consequences if left untreated. Moreover, since suicidal ideation is not limited to MDD but is common across severe psychiatric disorders, it may be a distinct symptom construct that embodies a specific type of pathological thinking that can be uniquely targeted by treatment. The approach of targeting symptom constructs that span beyond a specific diagnosis but have a well-defined neurobiology follows the research domain criteria (RDoC) defined by the National Institute of Mental Health^21^. As suicidal ideation is associated with impaired neuroplasticity^22^ and as ECT can effectively resolve suicidal ideation^3^, we sought to determine if production of neuroplasticity underlies the mechanism through which MST resolves suicidal ideation. Moreover, since our previous report showed that baseline GABAergic neurotransmission is associated with resolution of suicidal ideation following MST treatment^23^, a change in LICI that correlates with suicidal ideation reduction would further illustrate the importance of GABAergic inhibition for the therapeutic mechanism of MST. Since we expect MST to produce neuroplasticity (i.e., increased CEA) with decreased inhibition (i.e., decreased LICI), a reduction of suicidal ideation should be positively correlated with a decrease in LICI.

## Material and methods

### Participants

All patients included in this study participated in a larger open-label clinical trial of MST for severe psychiatric disorders at the Centre for Addiction and Mental Health (ClinicalTrials.gov Identifier: NCT01596608). All patients provided written informed consent. This study was approved by the ethics committee at the University of Toronto and Centre for Addiction and Mental Health. The patient sample was limited to those who had a diagnosis of MDD based on the Diagnostic and Statistical Manual of Mental Disorders (DSM-IV) and were deemed to be treatment resistant based on their medication history. Twenty-three patients (mean [SD] age: 45.0 [12.2] years, 11 males) with completed baseline and post assessments were part of this study. Demographic and clinical information is provided in Table 1.

**Table 1:** Demographics and clinical information

Category	Value
Sample size	23
Sex, no. (%)	
Male	11 (49)
Female	12 (51)
Age, mean (SD), years	45.0 (12.2)
Illness duration, mean (SD), years	20.7 (15.0)
No. of depressive episodes, mean (SD)^a^	8.3 (11.3)
ATHF (cumulative score for current episode), mean (SD)	11.6 (7.6)
Medication, *n*: dose (mg)	*Antidepressant*: Bupropion, 5: (300, 300, 300, 300, 450) Duloxetine, 4: (120, 60, 120, 120) Trazodone, 4: (50, 100, 50, 100) Desvenlafaxine, 3: (50, 150, 100) Escitalopram, 3: (40, 20, 35) Quetiapine, 3: (50, 300, 150) Fluoxetine, 2: (120, 60) Sertraline, 2: (100, 100) Mirtazapine, 1: (45) Nortriptyline, 1: (75) Venlafaxine, 1: (225) *Antipsychotic*: Aripiprazole, 2: (300, 10) Risperidone, 2: (2, 4) *Benzodiazepine:* Clonazepam, 5: (0.25, 1, 1, 0.5, 1) Lorazepam, 4: (1, 1.5, 0.5, 0.5) Zopiclone, 2: (7.5, 7.5) Diazepam, 1: (20) *Others*: Lithium, 1: (450) Pregabalin, 1: (150)

^a^Information was only available from 13 patients, 9 had too many episodes to keep track, and 1 patient had indeterminate number of episodes.

### MST treatment

MST stimulations were delivered with a MagPro MST machine (MagVenture) and a twin coil consisting of two individual circular coils symmetrically placed over the frontal cortex with the maximal electric field over the Fz electrode position. Treatments were administered under anesthesia and neuromuscular blocker for 24 sessions or until remission of depressive symptoms (defined as both a score of 10 or below and a 60% reduction in score on the 24-item Hamilton Rating Scale for Depression (HRSD) for at least 2 days after the last treatment). Further details of the treatment protocol and comprehensive clinical trial results will be reported in a separate manuscript currently in preparation.

### Suicidal ideation

Suicidal ideation was assessed with the Scale for Suicidal Ideation (SSI)^24^ at baseline and after the completion of the MST course.

### TMS-EEG testing

TMS-EEG measures were collected within 1 week before and within 2 days after the MST course. Single and paired pulse TMS stimulations (100 ms interstimulus interval) were delivered to the left DLPFC and to the left motor cortex as a control site. For each of the four conditions (i.e., DLPFC single pulse, DLPFC paired pulse, motor cortex single pulse, and motor cortex paired pulse), TMS stimulation was applied for 100 trials with a 70-mm figure-of-8 coil powered by two Magstim 200 stimulators joined together with a BiStim module. DLPFC stimulation was applied with the TMS coil centered over the F5 electrode and pointing forward along the line joining F5 and AF3. Motor cortex stimulation was applied at the optimal location for producing motor responses from the right abductor pollicis brevis (APB) muscle. TMS stimulation was administered at the intensity that produced a mean peak-to-peak motor-evoked potential of 1 mV from the APB muscle. While MRI guided stimulation would have been ideal, the nature of the test population, which include severe depression patients experiencing suicidal ideation, required that treatment start immediately and limited our ability to obtain MRIs for this study.

EEG was recorded with a 64-channel system (Neuroscan Synamps RT) and a cap placed in accordance to the international 10–20 system. To avoid EEG amplifier saturation and reduce the duration of TMS stimulation artifacts, the data were recorded in DC mode with a 20 kHz sampling rate and a 200 Hz low-pass filter (48 dB/octave).

### EEG preprocessing

Preprocessing of the EEG data was done in the same way as our previously published study^23^, which is briefly described here. Continuous EEG data were cut into 2-s trials with 1-s of data before and after the test stimulus onset (−1000 ms to 1000 ms). Each data trial was baseline corrected with the mean of the pre-stimulus period from −1000 ms to −110 ms. Data from 5 ms before to 10 ms after the onset of each TMS pulse were discarded to remove the large stimulation artifact. Trials and channels that were outliers in terms of amplitude or high-frequency power were removed through guided visual inspection. TMS decay artifacts were cleaned from the data through removal of characteristic noise components extracted with independent component analysis (ICA). After removal of any remaining outlier trials and channels, the data was low-pass filtered to the range of physiological EEG (i.e., 1–80 Hz) and a notch filter was applied to remove the line noise at 60 Hz. A second round of ICA was performed to identify and remove noise components such as eye blinks, eye movements, and muscle artifacts. Finally, any missing channels were interpolated and all channels were average referenced. The relevant scripts can be requested from the corresponding author, but the same processing steps are available through an expanded toolbox for TMS-EEG analysis^25^.

### TMS-EEG measures

**Fig. 1:**
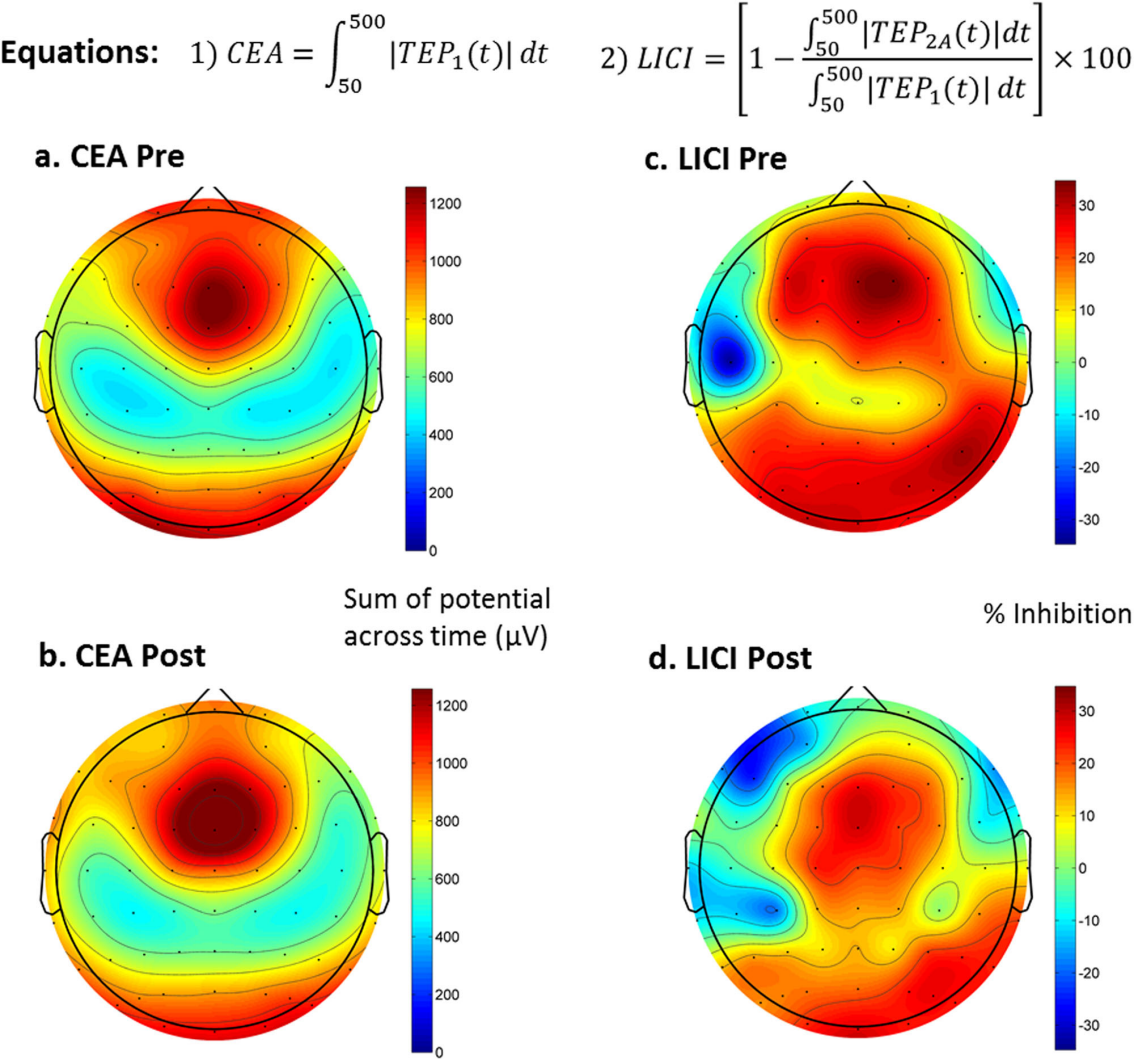
TMS-EEG measures of neuroplasticity and inhibition: cortical-evoked activity (CEA) was calculated using the single pulse TMS-evoked potential (TEP) in Eq. 1, while long-interval cortical inhibition (LICI) was calculated using both single and paired TEP in Eq. 2. Panels **a** and **c** show the baseline group average of the measures across all electrodes, while panels **b** and **d** show the post MST treatment group average of the same measures. The topoplots shown in this figure are all from the DLPFC condition. Please see Supplementary Fig. A1 for equivalent topoplots of the motor cortex condition

After preprocessing, EEG data trials were averaged for each recording condition of every patient to obtain TEPs, which are used to calculate the measures of CEA and LICI based on Eqs. 1 and 2, respectively (see Fig. 1). To simplify notation, we refer to the evoked response from a single pulse as TEP_1_ and that of a paired pulse as TEP_2_. TEP_2A_ refers to the adjusted paired pulse response and is calculated for each patient and stimulation region by subtracting TEP_1_ from TEP_2_ after aligning the onset of the test pulse for the former to the onset of the conditional pulse (i.e., first pulse) for the latter.

CEA was calculated as the area under the curve of the rectified TEP_1_ waveform across a certain time window based on previously published method^16^. The start and end points were chosen to be 50 and 500 ms in order to avoid any residual early artifacts and to capture the entire length of the post stimulus fluctuations. LICI was calculated as the percentage decrease in area under the curve of the rectified TEP_2A_ relative to the area under the curve of the rectified TEP_1_ in accordance with previously published method^26^. The window of analysis for LICI was also chosen to be 50–500 ms for better comparison with the CEA analysis.

### Statistical analysis

The clinical effect of MST on suicidal ideation was evaluated by comparing pre and post-treatment SSI scores with the Wilcoxon signed-rank test.

To assess the effect of MST on TMS-EEG measures of neuroplasticity (i.e., CEA) and cortical inhibition (i.e., LICI), a two-tailed paired *t*-test comparison of pre and post-treatment values was done for each electrode and corrected for multiple comparisons with cluster-based permutation analysis. Specifically, electrode *t*-scores with an associated *p*-value of ≤0.05 were clustered based on spatial adjacency. A cluster of electrodes is considered significant if its summed *t*-score is >95% of the values from a reference distribution consisting of maximum summed cluster *t*-scores. The reference distribution is generated by running the same paired comparison on 1000 simulated datasets created by randomly swapping pre and post-treatment values of patients.

In addition, correlations between changes in the TMS-EEG measures (i.e., CEA and LICI) and changes in the clinical variable (i.e., SSI score) were evaluated with the Spearman’s rank test. To control for multiple comparisons across electrodes, cluster correction was applied to the per electrode correlation values. Electrode correlation values associated with a *p*-value of <0.05 were clustered based on spatial adjacency. A cluster of electrodes is considered significant if its summed *t*-score is >95% of the values from a reference distribution consisting of maximum summed cluster *t*-scores. In this case, the reference distribution is generated by applying the same correlation analysis on 1000 simulated datasets created by permuting the subject label of the clinical values.

The effect of MST on each TMS-EEG measure (i.e., CEA and LICI) was also examined with a two-way analysis of variance (ANOVA) by dividing patients who had suicidal ideation at baseline (i.e., pre-SSI > 0) into two groups, those in whom suicidal ideation resolved (post-SSI = 0) or did not resolve (post-SSI > 0). The group was the between-subject variable, while time (pre vs. post treatment) was the within subject variable.

Finally, to assess whether changes in TMS-EEG measures can be used to identify those in whom suicidal ideation resolves, a logistic regression model was applied to electrodes for which there was a significant correlation between change in SSI score and change in TMS-EEG measure. The effectiveness of the classifier (i.e., TMS-EEG measure) was measured with the receiver-operating characteristic curve (ROC), which allows the sensitivity and specificity across different thresholds of the classifier to be evaluated. A larger area under the ROC curve indicates a better classifier.

## Results

### Clinical results

Based on the Wilcoxon signed-rank test, mean [±SD] SSI scores decreased significantly with MST (pre: 9.3 ± 6.3; post: 4.3 ± 5.6; *Z*-score = 3.44, *p* < 0.001). Out of 18 patients who had suicidal ideation at baseline, 8 were resolved of suicidal ideation (44.4%) while 10 were not (55.6%).

### CEA in the DLPFC

**Fig. 2:**
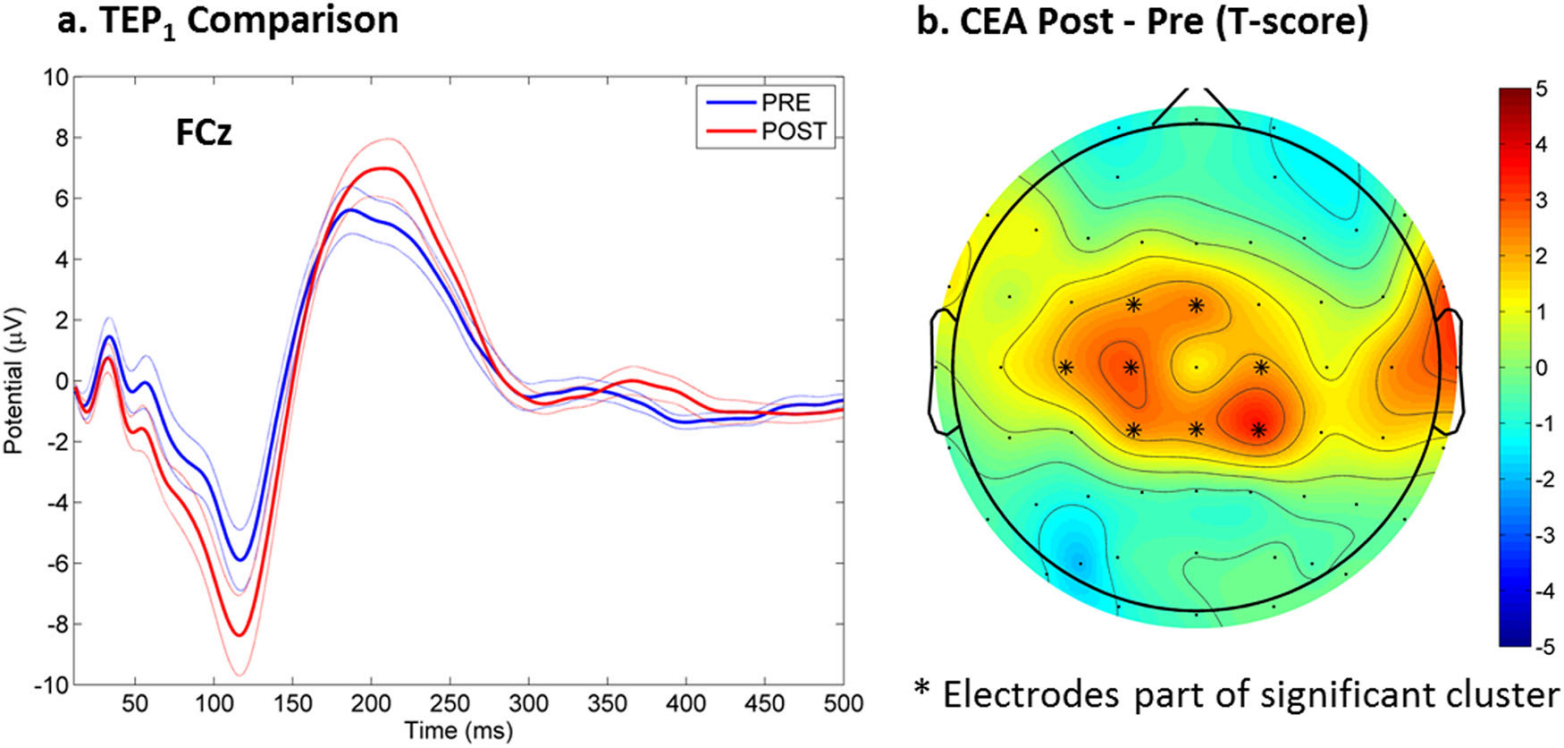
Changes in cortical-evoked activity (CEA) as a result of MST treatment: Panel **a** shows the single pulse TMS evoked potential (TEP) response at the FCz electrode as a result of DLPFC stimulation, both at baseline and after a course of MST treatment. Panel **b** shows the statistical result (*t*-scores) from comparing pre and post CEA values

Overall, there was a frontal central topography for CEA both pre- and post MST (see Fig. 1a, b). Averaging the trial responses of the single pulse condition generated a characteristic TEP (see Fig. 2a).

After MST, there was a significant increase in CEA over the frontal central electrodes (cluster *p* = 0.040) (see Fig. 2b).

There was no significant correlation between changes in CEA and changes in the SSI scores. The ANOVA analysis revealed no significant group effect or interaction.

### LICI in the DLPFC

**Fig. 3:**
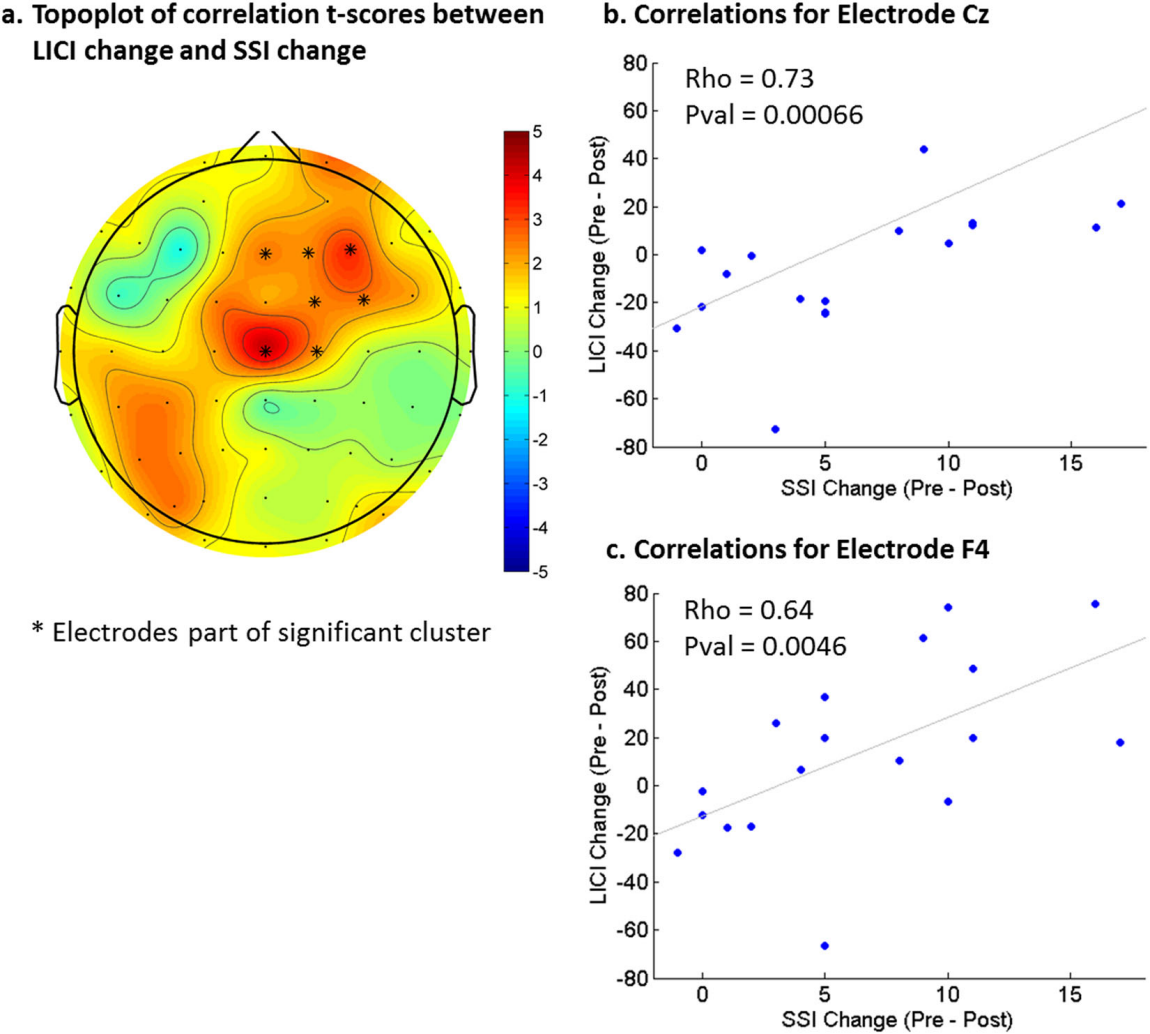
Correlation between change in long-interval cortical inhibition (LICI) measures and changes in suicidal ideation on the Scale for Suicidal Ideation (SSI): a higher decrease in SSI score is associated with a larger decrease in the LICI value from the DLPFC condition. The correlations are most significant over the right frontal cortex

**Fig. 4:**
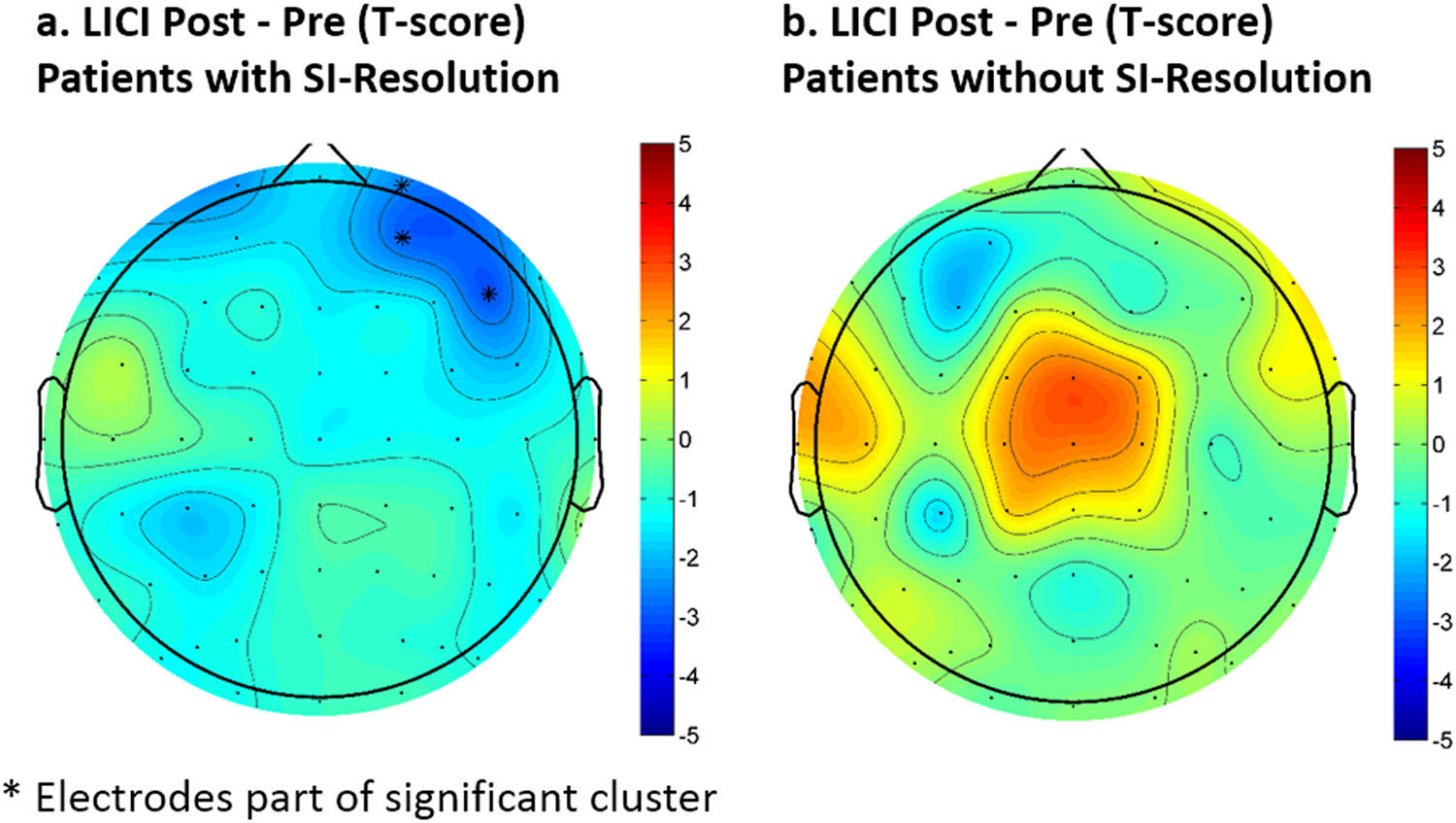
Changes in long-interval cortical inhibition (LICI) for the DLPFC condition as a result of MST treatment: Panel **a** shows the statistical result (*t*-scores) from comparing pre and post LICI values for patients with suicidal ideation resolution (SI-resolution), while panel **b** shows the same statistical comparison results for patients without SI-resolution. Patients in both groups had a Scale for Suicidal Ideation (SSI) score greater than zero at baseline, but patients with SI-resolution are those that have a zero SSI score after a course of MST treatment

As with CEA, the topography of LICI was frontal central (see Fig. 2c, d). There were no significant changes in LICI with MST but there was a significant correlation between the decrease in LICI over the frontal and central electrodes and the decrease in SSI scores (cluster *p* = 0.044, max at Cz, spearman rho = 0.73) (see Fig. 3). The ANOVA analysis revealed no significant group effect or interaction. However, it is worth noting that LICI was significantly decreased after MST over the right frontal electrodes (Fig. 4) when the patients were limited to only those who were resolved of suicidal ideation after MST (cluster *p* = 0.048, *n* = 8). No significant LICI change was found when patients were limited to only those who were not resolved of suicidal ideation after MST.

### CEA and LICI in the motor cortex

The topography of CEA and LICI was frontal central, both pre- and post MST. There was no significant change in CEA or LICI with MST, no significant correlations between changes in these measures and changes in SSI scores, and no group effect or interaction with the ANOVA (see Supplementary Fig. A1).

### Binary classification analysis

**Fig. 5:**
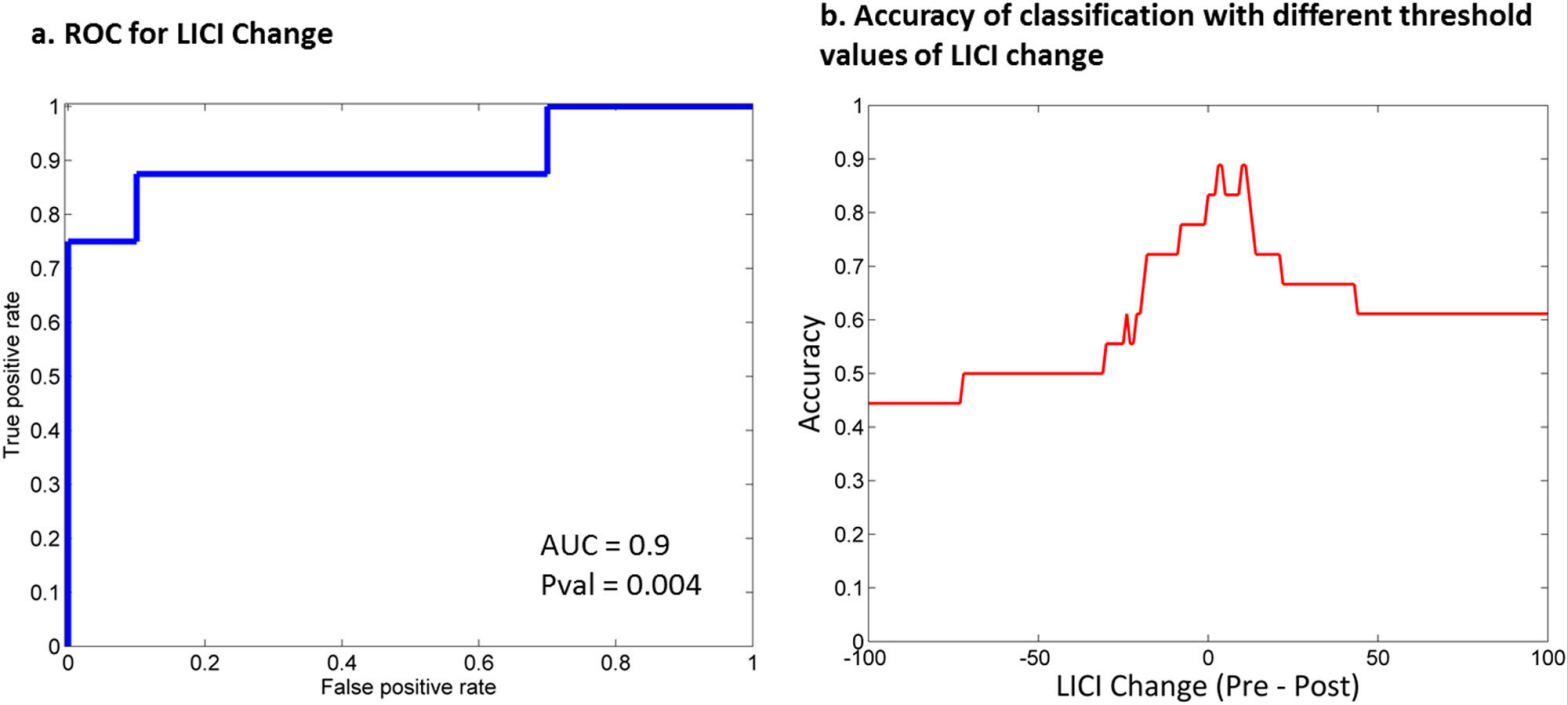
Identifying patients in whom suicidal ideation resolved using changes in long-interval cortical inhibition (LICI) from the DLPFC condition: receiver-operating characteristic (ROC) curve for identifying resolution of suicidal ideation (defined as having a pre-treatment score of 1 or above and a post-treatment score of 0 on the Scale for Suicidal Ideation (SSI). Panel **a** shows the ROC curve with LICI change as the classifier. Panel **b** shows the plot for accuracy of prediction based on different threshold values of LICI change

Changes in LICI of the DLPFC condition can be used to classify those patients experiencing or not a resolution of their suicidal ideation as a result of MST. The electrode showing the best classification result is Cz, with a 90% sensitivity and 88% specificity (AUC = 0.9, *p* = 0.004) (see Fig. 5). Data obtained during the TMS stimulation of the motor cortex could not be used to identify those experiencing a resolution of their suicidal ideation.

## Discussion

This study demonstrates that MST produced significant resolution of suicidal ideation. MST also produced neuroplasticity in the brain. That is, MST increased CEA in the prefrontal cortex but not in the motor cortex of patients with TRD. MST also decreased cortical inhibition (i.e., decreased LICI), which was greatest in patients with the largest reduction in suicidal ideation. The reduction in LICI can identify patients achieving resolution of suicidal ideation with 90% sensitivity and 88% specificity. The observed increase in CEA and concomitant correlation of LICI decrease with suicidal ideation reduction suggest that MST may achieve its therapeutic effects through improved LTP-like plasticity. The significance of these findings will be discussed below.

With 8 out of 18 patients (44%) completely resolved of suicidal ideation, MST can be considered highly effective for reducing suicidal ideation. This is especially evident in comparison to the minimal reduction in suicidal ideation with antidepressant medication^27^, which has actually been associated with increased suicidal behavior based on earlier studies. However, the clinical benefit of MST may be less in comparison to ECT, which has been shown to produce resolution of suicidal ideation in 80% of patients after a course of treatment based on a subscale of the HRSD scale^3^. A future study is necessary to compare the efficacy of MST relative to ECT under the same control conditions and with the same rating scale.

Previous studies have demonstrated that increased CEA is related to LTP-like plasticity in the cortex^16,28^. Our result of increased CEA after MST is in agreement with previous literature and supports the hypothesis that MST works by producing LTP-like plasticity. Given the complex nature of the TEP waveform, an increase in CEA suggests an overall production of neuroplasticity due to reconfigurations of both excitatory and inhibitory connections. Such reconfigurations can be the result of larger brain networks being engaged by stronger connections from the site of stimulation or improved synchrony within the recruited neuronal population^29^. The reconfiguration process is likely dependent on the parameters of the initial MST stimulus, but is amplified spatially and is maintained by the ensuing seizure, which can synchronize both cortical and subcortical regions spanning the entire brain^30^. From this perspective, LTP-like plasticity produced by MST is likely achieved through both homosynaptic and heterosynaptic mechanisms. Specifically, the initial stimulus represents a tetanic entrainment of local neurons (i.e., homosynaptic), while the resulting seizure allows many neurons across the brain to interact synchronously (i.e., heterosynaptic). Indeed, the transient and unnaturally high level of synchrony reached during a seizure has a strong ability to modify synaptic connections between neurons and promote the growth of new and existing neurons^31^. Since the change in CEA was only significant in the DLPFC and not in the motor cortex, our results also suggest that MST may have produced network specific neuroplasticity. That is, only the network engaged by activating the DLPFC was reconfigured by the MST treatments.

Several lines of evidence suggest that seizure therapy (i.e., ECT or MST) produces neuroplasticity. Anatomically, seizure therapy is associated with increased thickness of the prefrontal and cingulate cortices^12^, larger volumes for the hippocampus and amygdala^11^, and improved integrity of white matter tracts for the anterior cingulum^13^. Evidence for neuroplasticity at the cellular level involves both neurons and cells that support neuronal function. One crucial finding is that adult hippocampal neurogenesis is impaired with MDD and improves after effective treatments^32^. MDD is also associated with impaired gliogenesis across several brain regions, including the prefrontal cortex^33^ and anterior cingulate cortex^34^. These deficits may be rectified by seizure therapy, as demonstrated by animal models showing increased neurogenesis^35^ and gliogenesis^36^ after electroconvulsive seizures. Finally, MDD is also associated with decreased neurotrophic signaling, especially for the brain-derived neurotrophic factor (BDNF)^37^, while electroconvulsive seizures can lead to an upregulation of neurotrophic signaling^38^.

The association between antidepressant treatments and production of neuroplasticity is not limited to seizure therapy; it has also been demonstrated with antidepressant medications^39^, exercise^40^, and light therapy^41^. In fact, neuroplasticity, in particular hippocampal neurogenesis, may be fundamental to treating depression^42^. Future studies are needed to compare the extent to which different depression treatments (i.e., psychotherapy, antidepressant medications, or brain stimulation) affect neuroplasticity as differences may explain variability in therapeutic efficacy.

We also found that there was a significant correlation between decreases in LICI and reduction in suicidal ideation following MST treatment. This result suggests that the variation in response to MST may be driven by individual differences in GABAergic inhibitory network activity. Our previous results in a partially overlapping sample have shown a correlation between baseline GABAergic measures (e.g., LICI) and changes in suicidal ideation as a result of MST^23^. Our current result extends those findings by showing that a decrease in GABAergic inhibition, specifically as indexed by LICI, is associated with a decrease in suicidal ideation reduction. However, we did not find a significant change in LICI after MST on a group level, which is likely due to heterogeneity in the patients’ response to the treatment. Indeed, by examining the scatter plots shown in Fig. 3, one can see that some patients had an increase in LICI while others had a decrease in LICI after the treatment. Moreover, when examining only patients who were resolved of suicidal ideation, there was a significant decrease in LICI over the right frontal electrodes as a result of MST (Fig. 4). Overall, evidence from this study suggests that GABAergic inhibition may be a biological substrate that underlies the therapeutic change in patients and also supports the importance of the GABAergic system in the pathophysiology of suicidality as demonstrated by previous studies^43^.

Our finding of concurrent production of neuroplasticity and decreased inhibition is in line with the mechanism of LTP-like plasticity. For example, it was demonstrated that ischemic nerve block of the forearm during rTMS stimulation of the contralateral motor cortex leads to a simultaneous decrease in inhibition and an increase in neuroplasticity of the cortex^44^. Along these lines, other studies reported that ischemic nerve block during motor practice can also lead to an increase in neuroplasticity, which is reduced when pretreated with a GABA acting drug, lorazepam^45^. Finally, in a study with magnetic resonance spectroscopy, it was found that a decrease in GABA concentrations in the motor cortex as a result of transcranial direct current stimulation predicted better motor learning during a force adaptation task^46^. Our clinical findings are also in line with animal model research showing that suppression of GABAergic inhibition leads to improved LTP^19^. Given the concurrent increase in CEA after treatment, the correlation between LICI change and suicidal ideation reduction suggests that individual specific reconfiguration of the frontal GABAergic network is necessary for improved LTP of the brain. Evidence for this mechanism is supported by that of ketamine, a rapid acting antidepressant that has also been shown to produce suicidal ideation reduction^47^. Indeed, it has been suggested that ketamine may work in part via inhibition of tonically active GABAergic interneurons, which leads to disinhibition of glutamate signaling and ultimately synaptogenesis in a similar mechanism as LTP^48^.

Our binary classification results showed that changes in LICI can identify patients who experience resolution of their suicidal ideation from those who do not with 90% sensitivity and 88% specificity. While a measure of change associated with treatment cannot be used to predict treatment outcome, this result provides some insight into the mechanism of MST. In particular, MST may have a dichotomous effect on GABAergic inhibition that underlies the binary outcome for resolution of suicidal ideation. Our previous study demonstrated that baseline LICI is correlated with the change in suicidal ideation, but the measure is only useful in predicting the resolution of suicidal ideation as a supplement to another predictor (i.e., N100)^23^. In comparison, the change in LICI found in this study was able to identify patients in whom suicidal ideation resolved with reasonable accuracy when used alone. This means that the change in cortical inhibition as captured by LICI may be important to the neuroplasticity effect of MST.

Our study has some limitations. First, our pilot TMS-EEG study of MST treatment had a small sample (*N* = 23). A larger sample is needed to confirm our findings. Second, future studies will need to compare MST with another form of treatment, to distinguish the specific effects of MST from that of antidepressant treatments in general. Third, as in all clinical human studies, our neurophysiological measures are indirect. The proposed mechanism of MST should be verified in animal models where changes in specific neuronal features (e.g., number and morphology of GABAergic neurons) can be quantified directly.

In conclusion, our study suggests that MST produces neuroplasticity in the frontal cortex through LTP-like mechanisms, which occurs in tandem with individual specific reductions in prefrontal GABAergic inhibition that are indicative of suicidal ideation resolution after treatment. These findings may point to better ways of optimizing treatments for patients with TRD while improving our understanding of the biological mechanisms behind the disorder.

## Electronic supplementary material

Supplementary Figure
