# Supplementary material for: Magnetic seizure therapy reduces suicidal ideation and produces neuroplasticity in treatment-resistant depression
Source: Transl Psychiatry. 2018 Nov 23;8:253. doi: 10.1038/s41398-018-0302-8 (PMC6251931; doi:10.1038/s41398-018-0302-8)
Supplement: Supplementary file 1 — Supplementary Figure [file 41398_2018_302_MOESM1_ESM.docx]

**Supplementary Figure**


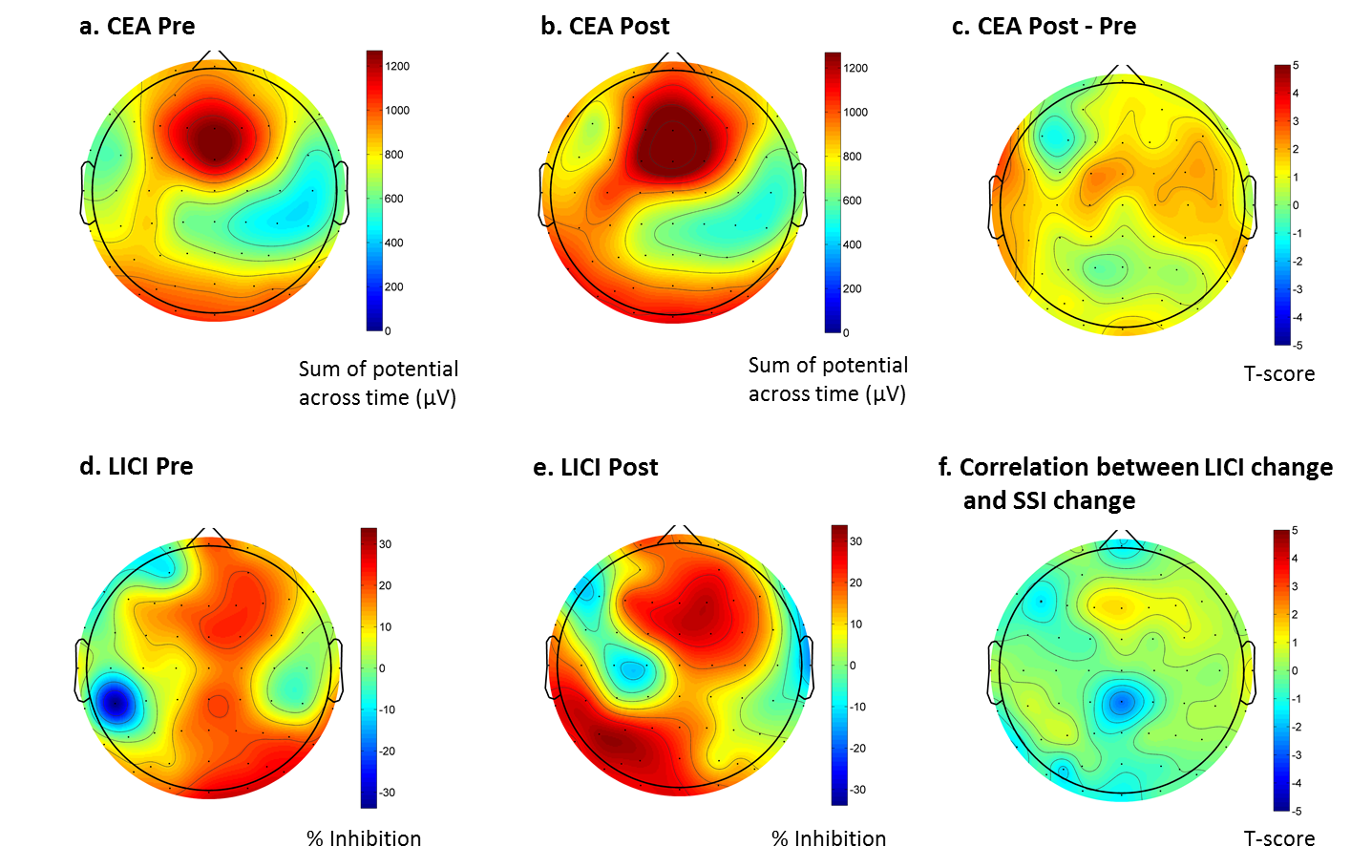


**Fig A1.** TMS-EEG measures of the motor cortex condition and results from relevant statistical analysis: Cortical Evoked Activity (CEA) was calculated using the single pulse TMS evoked potential (TEP) in equation 1, while Long-Interval Cortical Inhibition (LICI) was calculated using both single and paired TEP in equation 2. Panels a) and b) shows the baseline group average of the measures across all electrodes; panels c) and d) shows the post MST treatment group average of the same measures; panel e) shows the statistical result (t-scores) from comparing pre and post CEA values; panel f) shows the correlation (t-scores) between the change in LICI and change in suicidal ideation on the Scale for Suicidal Ideation (SSI).
